# Normative Hand Strength Values for the Healthy Adult Trinbagonian Population and Comparison to International Data

**DOI:** 10.7759/cureus.67230

**Published:** 2024-08-19

**Authors:** Emerson Budhoo, Saeed R Mohammed, Akisha Glasgow, Haroun Choate, Rei S Medford, Abigail Cooblal, Kristin Fung, Akilah Cateau, David R Santana, Marlon M Mencia, David Deane, Paula Kassie, Dale Maharaj

**Affiliations:** 1 Orthopedics, Eric Williams Medical Sciences Complex, Champ Fleurs, TTO; 2 Clinical Medical Sciences, The University of the West Indies - St. Augustine Campus, St. Augustine, TTO; 3 Oncologic Surgical Pathology, Memorial Sloan Kettering Cancer Center, Manhattan, USA; 4 Mathematics and Statistics, The University of the West Indies - St. Augustine Campus, St. Augustine, TTO; 5 Pediatric Surgery, Eric Williams Medical Sciences Complex, Champ Fleurs, TTO; 6 Sports and Exercise Medicine, Trinidad and Tobago Police Service, Port of Spain, TTO; 7 Orthopedics, Arima District Health Facility, Arima, TTO; 8 Orthopedics, Sangre Grande Hospital, Sangre Grande, TTO; 9 Clinical Surgical Sciences, The University of the West Indies - St. Augustine Campus, St. Augustine, TTO; 10 Orthopedic Surgery, Eric Williams Medical Sciences Complex, Champ Fleurs, TTO; 11 Vascular Surgery, Caribbean Vascular and Vein Clinic, Port of Spain, TTO

**Keywords:** orthopedics, palmar pinch, key pinch, public health, surgery, hand grip

## Abstract

Introduction: It has been well established that grip strength measurements can be useful as a benchmark for comparing the efficacy of different treatment modalities as well as an aid in the assessment of the progress of disease and rehabilitation. Grip strength has also been shown to be a representative marker for sociodemographic factors.

Methods: Participants were selected from five different regions in a cross-sectional manner from the streets of Trinidad and Tobago, and a Jamar hand dynamometer was used to assess the metrics of hand grip, palmar grip, tip pinch, and key pinch across both hands. Data was analyzed comparing right and left as well as dominant and non-dominant hands, and participants were classified by occupation.

Results: We enrolled 1233 participants in this study, of which the majority were female (54.5%). 90% of participants were right-hand dominant. The mean hand strength of the dominant hand was significantly greater than the non-dominant for all four strengths assessed. Participants of Afro-Trinbagonian descent were shown to have the highest mean values overall. We found no significant relationship between occupational intensity and mean grip strengths. For the male population, it was found that height, age, and BMI were all significant predictors of hand strength. This was notably only so for a minority of the female population tested.

Conclusion: This study serves to provide the normative data for the adult healthy Trinbagonian population. Further research to determine better predicative variables specific to the female population is needed.

## Introduction

Normative hand strength values are of both clinical and legal significance. Previous studies have demonstrated that grip strength can (1) function as a measure for comparison of surgical procedures/techniques, (2) facilitate assessment of rehabilitation therapy, and (3) allow for evaluation of disability and recovery after injury/impairment from musculoskeletal, cerebrovascular, neuromuscular, and cardiorespiratory diseases/conditions [1-6]. Grip strength has also proven to be predicative of outcomes such as mortality and disability and may be associated with health-related quality of life [7,8]. This has led numerous authors to recommend regular use of grip strength as a screening method in primary and tertiary care [7,9].

The hand is a highly complex motor organ with a myriad of functions, and as such, objective assessment of its function should encompass testing of both the intrinsic and extrinsic muscles. It is well established that hand strength correlates with sociodemographic factors such as height, weight, ethnicity, sex, and occupation [10,11]. 

Normative data for hand strength in the Trinbagonian population has not been documented/published previously. This study was designed primarily to establish the normal/baseline values of hand strength (comprised of hand grip strength, palmar grip strength, key pinch strength, and tip pinch strength) in the healthy population of Trinidad and Tobago. A secondary objective was to assess the relationship between these strengths and demographic variables. The results of our population were further compared to international data.

## Materials and methods

Ethical approval and study design

Ethical approval was obtained from the University of the West Indies prior to the start of this study. The study was conducted in accordance with the declaration of Helsinki, 1964. Willing persons were informed about the study protocol and procedures of the study and obtained written informed consent from each person who agreed to participate in the study. The confidentiality of all participants was maintained throughout the study and thereafter.

A cross-sectional analysis was performed across five major cities/towns in Trinidad and Tobago, one per each Regional Health Authority. Simple random sampling methods were employed on sidewalks in these regions.

Study participants

**Figure 1:**
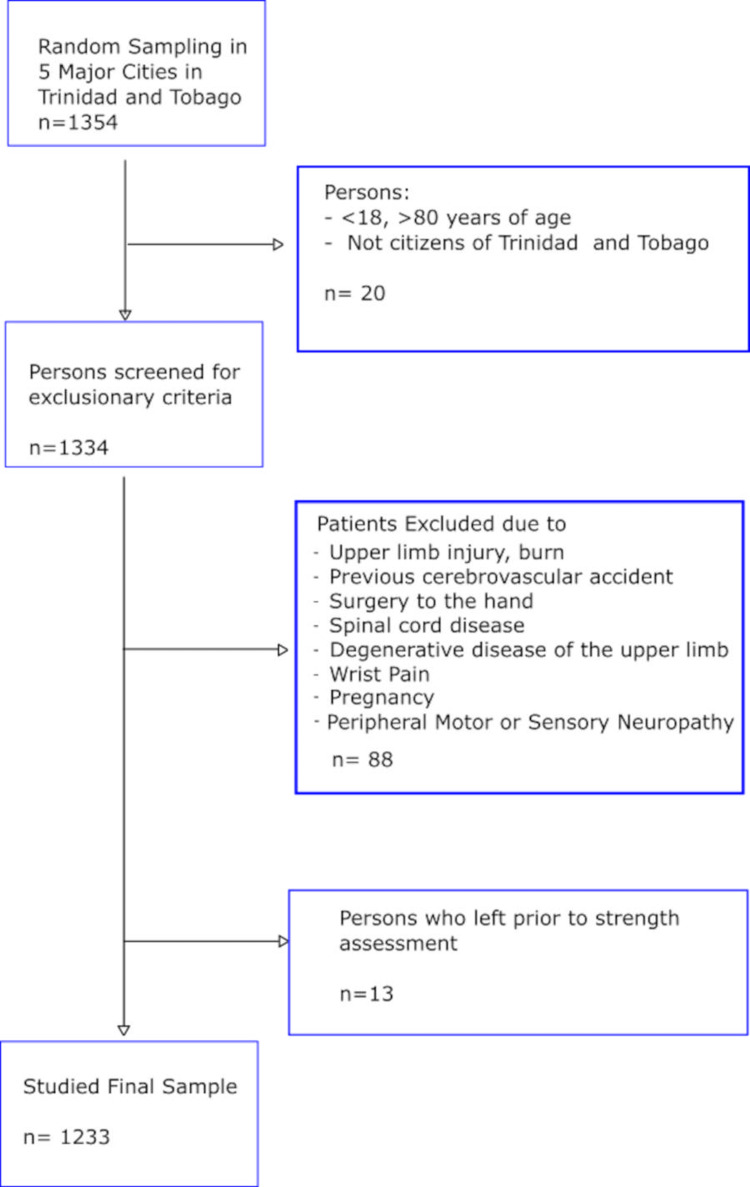
Flow chart displaying inclusion and exclusion criteria

The study included 1354 adult subjects. The inclusion and exclusion criteria for this study are detailed in Figure 1. 1233 participants were ultimately included for data analysis.

Procedures

The height (in centimeters) and weight (in kilograms) of each participant were assessed, utilizing a standardized scale; the same brand and model were used at all five locations. The Jamar Hand Dynamometer was then used to assess hand grip, palmar grip, tip pinch, and key pinch strengths for both the left and right hands. Three consecutive trials were performed for each test, at one-minute intervals, and the mean value of these was used for statistical analysis. The dominant hand was defined as the one that was used for writing. Occupation was classified according to the International Standard Classification of Occupations (ISCO): (1) managers, (2) professionals, (3) technicians and associate professionals, (4) clerical support workers, (5) service and sales workers, (6) skilled agricultural, forestry, and fishery workers, (7) craft and related trades workers, (8) plant and machine operators and assemblers, (9) elementary occupations, and (10) armed forces occupations [12].

Statistical analysis

Statistical analysis of the data was accomplished by using SPSS. Frequencies were used to summarize the nominal data. Means and standard deviations (SD) were calculated for continuous data.

ANOVA was used to identify if there were significant differences in grip strengths for gender groups, ethnicity groups, and occupation groups.

Additionally, linear regressions were conducted for males and females for all grip strength measures, with age, weight, height, and BMI being used as independent variables. The results of these regressions are presented in the relevant section.

## Results

Overall, 1233 participants were included in the data analysis (Figure 1); 561 (45.5%) males and 672 (54.5%) females. The mean height, weight, and BMI for males were 1.75+0.086 (m), 81.2+17.0 (kg), and 26.2±(5.16), respectively, and 1.63±0.080 (m), 74.8±18.1 (kg), and 28.1±6.50 for females. Most participants (90%) were right-hand dominant. Participants were of Afro-Caribbean (51.4%), Indo-Caribbean (25.4%) and mixed (23.2%) ethnicity. For all grip strengths, there was a significant difference between males and females. Table 1 displays the demographic data of the population and mean hand strengths, stratified by sex.

**Table 1:** Demographic information of the study population and mean hand strengths, stratified by sex

Characteristic	Male	Female
Number	561	672
Height, m	1.75 (±0.086)	1.63 (±0.080)
Weight, kg	81.2 (±17.0)	74.8 (±18.1)
BMI	26.2 (±5.16)	28.1 (±6.50)
Mean grip strength (dominant hand), kg		
Hand grip	23.6 (±10.6)	12.6 (±6.06)
Tip pinch	4.98 (±2.19)	3.61 (±1.56)
Key pinch	7.37 (±3.15)	4.97 (±2.05)
Palmar grip	6.21 (±2.61)	4.49 (±1.88)
Mean grip strength (non-dominant hand), kg		
Hand grip	23.1 (±10.7)	12.0 (±5.61)
Tip pinch	4.71 (±2.09)	3.29 (±1.41)
Key pinch	6.89 (±2.95)	4.55 (±1.89)
Palmar grip	5.87 (±2.53)	4.18 (±1.78)

Tables 2-5 demonstrate the mean strengths of the dominant, non-dominant, right, and left hands in men and women, for all four grips, stratified into age groups. The male sex was associated with higher mean strength for all four grips assessed when compared to the female sex (p<0.001).

**Table 2:** Mean hand grip strength in kilograms for men and women, presented in ascending age groups R, right; L, left; D, dominant; ND, non-dominant

Age group	Male				Female			
	R	L	D	ND	R	L	D	ND
<19	22.9	23.1	20.3	20.9	13.32	12	13.9	12.7
20-24	25.3	25.1	23.6	22.2	12.5	11.9	11.3	10.8
25-29	23.6	24	21.6	21.2	13.6	13.2	12	11.2
30-34	25.1	25.3	23	20.9	13.3	12.4	12.4	11.3
35-39	24.1	24.9	23.6	23.6	12.9	12.7	11.4	11.1
40-44	25.8	26.6	24.2	23.9	14.1	13.2	12.4	12.1
45-49	23	23.5	18.8	19.4	12.8	12.6	12	11.5
50-54	22.9	22.1	19.5	18.6	12.7	12	11.3	11.3
55-59	20.4	19.7	16.9	17.2	12.4	12.5	11	11.5
60-64	21.4	21	20.6	19	10.7	10.8	10	9.8
65-69	19.8	20.1	20.6	19.3	9.9	10.2	9.4	8.9
70-74	19.7	19.7	21.5	18.8	8.7	8.4	8.4	8.5
75-79	21.2	19.1	19.7	18.9	6.7	7.2	7.1	7.2
80+	17.4	17.8	17.4	17.8				

**Table 3:** Tip pinch grip strength in kilograms for men and women, presented in ascending age groups R, right; L, left; D, dominant; ND, non-dominant

	Tip pinch							
Age group	Male				Female			
	R	L	D	ND	R	L	D	ND
<19	4.23	4.08	4.63	4.35	3.63	3.63	3.59	3.18
20-24	4.54	4.08	4.9	4.71	2.945	2.72	3.31	3.12
25-29	3.93	3.78	4.73	4.42	3.18	2.87	3.63	3.36
30-34	5.44	4.69	5.27	5.02	2.87	2.72	3.55	3.15
35-39	4.08	4.38	4.85	4.76	3.02	2.57	3.46	3.15
40-44	4.38	4.38	5.07	4.78	3.93	3.33	3.97	3.55
45-49	4.23	3.93	5.02	4.74	2.87	3.33	3.77	3.6
50-54	4.84	4.54	5.31	5.03	3.63	3.18	3.8	3.34
55-59	4.23	3.48	4.68	4.35	3.18	2.87	3.5	3.19
60-64	5.14	4.46	5.11	4.71	3.02	2.72	3.47	3.12
65-69	5.75	5.44	5.31	5.14	3.78	3.48	3.65	3.46
70-74	4.84	3.86	4.83	5.12	2.795	2.645	2.98	2.77
75-79	5.29	4.38	4.95	4.64	3.025	2.8	3	2.89
80+	3.1	3.25	3.1	3.25				

**Table 4:** Key pinch grip strength in kilograms for men and women, presented in ascending age groups R, right; L, left; D, dominant; ND, non-dominant

Key pinch								
Age group	Male				Female			
	R	L	D	ND	R	L	D	ND
<19	6.5	5.9	6.93	6.61	3.63	3.63	5.47	4.82
20-24	6.96	6.96	8.04	7.53	2.945	2.72	4.82	4.45
25-29	6.35	5.75	7.31	6.86	3.18	2.87	5.2	4.84
30-34	7.11	6.8	7.69	7.28	2.87	2.72	5.12	4.67
35-39	6.2	6.35	7.54	7.25	3.02	2.57	4.89	4.54
40-44	6.5	6.125	7.67	7.19	3.93	3.33	5.42	4.85
45-49	5.75	5.44	7.07	6.6	2.87	3.33	4.99	4.67
50-54	6.35	5.745	7.45	6.88	3.63	3.18	5.16	4.61
55-59	5.37	4.99	6.76	6.28	3.18	2.87	4.67	4.32
60-64	6.425	5.365	6.78	6.19	3.02	2.72	4.66	4.14
65-69	7.71	6.96	7.4	6.81	3.78	3.48	4.58	4.4
70-74	6.955	6.5	6.99	6.04	2.795	2.645	3.69	3.66
75-79	7.56	5.75	6.76	5.81	3.025	2.8	4.18	3.63
80+	4.54	4.16	4.54	4.16				

**Table 5:** Palmar pinch grip strength in kilograms for men and women, presented in ascending age groups R, right; L, left; D, dominant; ND, non-dominant

Palmar pinch								
Age group	Male				Female			
	R	L	D	ND	R	L	D	ND
<19	5.75	4.84	5.97	5.76	4.54	4.54	4.95	4.43
20-24	6.35	6.05	6.56	6.34	3.405	3.48	4.42	4.1
25-29	4.91	4.38	5.98	5.52	4.005	3.63	4.5	4.34
30-34	6.35	6.2	6.43	6.2	3.55	3.18	4.51	4.06
35-39	5.37	5.22	6.25	6.05	3.63	3.44	4.34	3.95
40-44	5.365	5.215	6.78	6.26	4.08	3.78	4.92	4.57
45-49	5.065	4.99	5.89	5.64	3.855	3.745	4.6	4.36
50-54	5.215	4.84	6.3	5.83	4.54	3.93	4.69	4.27
55-59	4.69	4.31	5.75	5.36	3.63	3.48	4.33	4
60-64	5.515	5.515	5.83	5.57	3.63	3.63	4.19	3.98
65-69	6.96	6.35	6.49	6.16	4.38	3.855	4.13	4.02
70-74	5.97	5.515	6.07	5.25	3.18	2.87	3.39	3.47
75-79	6.05	5.14	5.85	5.23	3.78	3.78	3.88	3.75
80+	4.54	3.78	4.54	3.78				

**Figure 2:**
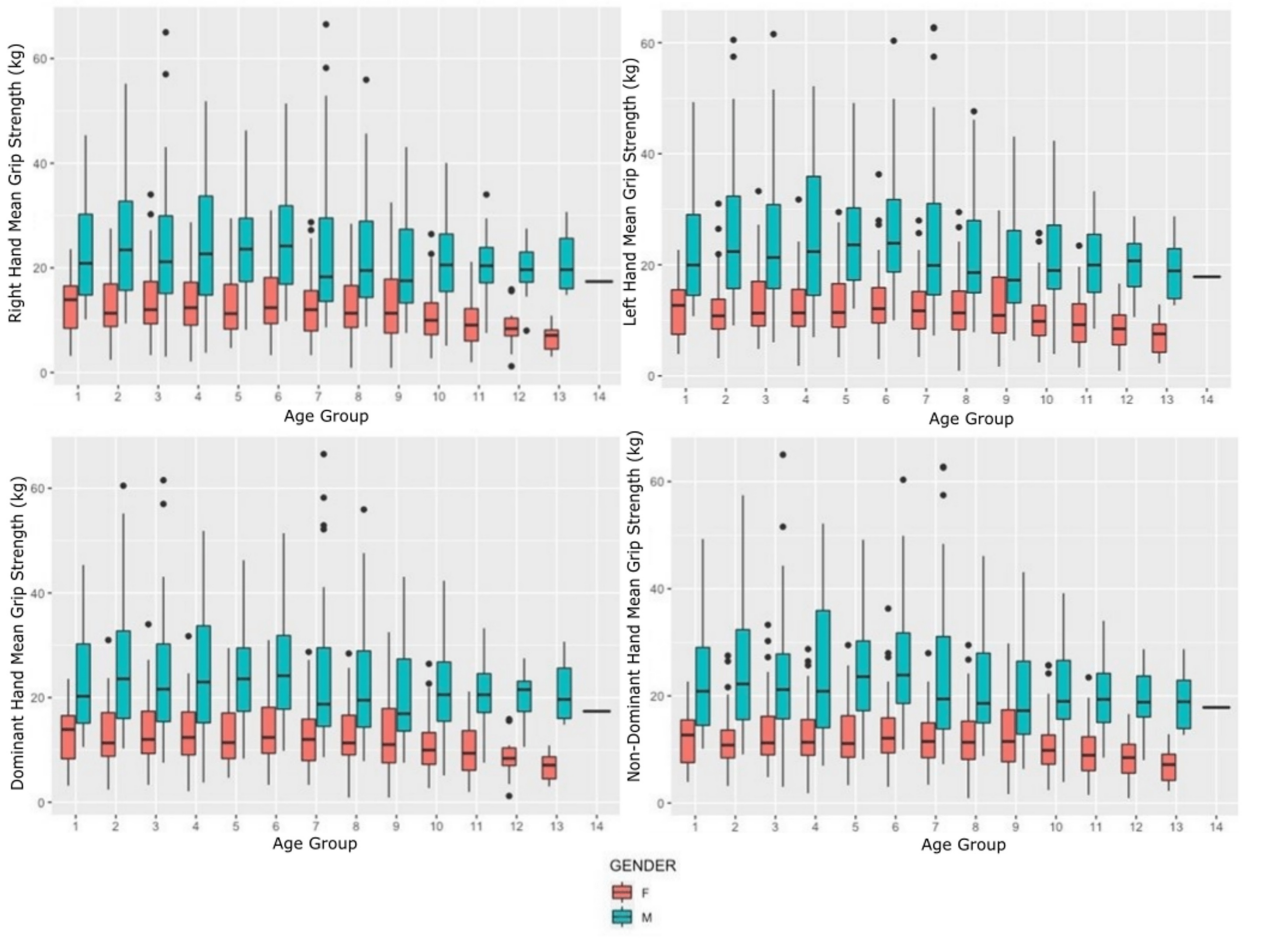
Box plots of the hand-grip strength measurements (kilograms) for the right, left, dominant, and non-dominant hands in men and women For each box, the center line represents the median, the height of the box represents the interquartile range, and the error bars represent minimum and maximum values.

The mean hand strength of the dominant hand was significantly greater than that of the non-dominant hand (p<0.001) for all four strengths assessed across the population (Tables 2-5). Hand strength was also compared between men and women (Table 6, Figure 2).

**Table 6:** Comparison of mean dominant and non-dominant grip strength for men and women D, dominant; ND, non-dominant

Male												
Hand grip			Tip pinch			Key pinch			Palmar pinch			
D	ND	%	D	ND	%	D	ND	%	D	ND	%	
23.59	23.07	97.78	4.98	4.71	94.58	7.37	6.88	93.34	6.21	5.87	94.48	
Female												
Hand grip			Tip pinch			Key pinch			Palmar pinch			
D	ND	%	D	ND	%	D	ND	%	D	ND	%	
12.65	11.99	94.86	3.61	3.29	91.16	4.97	4.55	91.53	4.49	4.18	93.08	

The mean values for the four grips assessed were quite different for the three ethnic groups (Table 7). Participants of Afro-Caribbean descent had higher mean values than those of mixed descent, while those of Indo-Caribbean descent recorded the lowest mean values. When accounting for categorical variables, ethnicity was a significant factor in both the dominant and non-dominant hands for hand grip and key pinch strengths (p<0.001) but was not associated with tip pinch or palmar grip strengths.

**Table 7:** Mean grip strength in kilograms of each grip type across ethnicity R, right; L, left; D, dominant; ND, non-dominant; A, African; I, East Indian; M, mixed

Ethnicity	Hand grip			
	R	L	D	ND
A	18.54	18.25	18.74	18.06
I	15.38	15.48	15.56	15.29
M	17.21	16.91	17.44	16.68
	Tip pinch			
	R	L	D	ND
A	4.36	4.05	4.36	4.04
I	4.09	3.82	4.08	3.83
M	4.11	3.82	4.11	3.81
	Key pinch			
	R	L	D	ND
A	6.36	5.88	6.36	5.87
I	5.61	5.23	5.61	5.23
M	5.91	5.46	5.91	5.46
	Palmar grip			
	R	L	D	ND
A	5.38	5.04	5.39	5.03
I	5.18	4.96	5.17	4.97
M	5.11	4.75	5.12	4.73

Each dominant hand grip strength measure was considered as a dependent variable for further statistical analyses utilizing linear regression, which tested whether age, weight, height, and BMI significantly predicted hand strength in both sexes. The fitted regression models are displayed in Table 8.

**Table 8:** Fitted regression models for dominant hand strength measures in men and women

Grip	Men	Women
Hand grip	-0.094 (age)+11.721 (height)+0.262 (BMI) |R^2^=84.1%	-12.384-0.047 (age)+16.61 (height) |R^2^=7.2%
Palmar grip	3.519 (height) |R^2^=85.0%	Neither age, weight, height, nor BMI were found to be significant
Tip pinch	1.875 (height)+0063 (BMI) |R^2^=83.9%	Neither age, weight, height, nor BMI were found to be significant
Key pinch	3.155 (height)+0.069 (BMI) |R^2^=84.9%	5.501-0.012 (age) | R^2^=0.8%

Participants were classified according to the ISCO and then stratified these occupations into three categories: low intensity (no occupation, i.e., homemakers, self-employed persons, students, and retirees), medium intensity (ISCO categories 1-4), and high intensity (ISCO categories 0, 5-9). Some participants did not provide an occupation and were thus excluded from this portion of the analysis. There was no significant relationship between occupational intensity and mean grip strengths.

**Figure 3:**
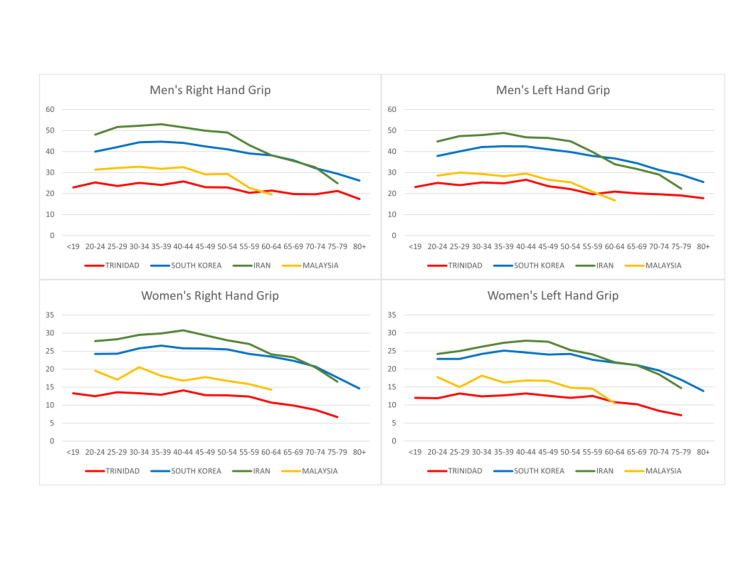
Comparison of the Trinbagonian population and international norms for men and women

It is well understood that hand strength varies significantly among populations. The mean hand grip strength for our population was thus compared to normative hand grip strength values from South Korean, Iranian, and Malaysian populations (Figure 3) [13-15].

## Discussion

The primary aim of this study was to provide normative values for hand strength in the Trinbagonian population. This would serve as a general guide for clinicians when assessing patients. Hand strength in our population varies significantly from that of the population(s) from South Korea, Iran, and Malaysia (Figure 3). 

The mean values for hand strength in the dominant and non-dominant hands in this study were comparable, with the mean non-dominant hand strengths exceeding 90% of the value of the dominant hand, for all four grips and across both sexes. We classified hand dominance according to the hand the participant wrote with, but several participants noted they were "born" left-handed but were forced to "change" to right-handed during early childhood. Similarly, others stated that although they wrote left-handed, they used their right hand for the performance of strenuous activities and for activities that required fine motor skills/coordination. Historically, the "10% rule" has been used in hand rehabilitation; this rule states that the dominant hand possesses 10% greater strength than the non-dominant hand. The results from this study indicate that this 10% difference may be exaggerated (Table 6).

Hand strength is influenced by factors such as a person’s sex, age, height, weight, ethnicity, and occupation, and hand factors such as the length and width of the palm and digit ratios [16]. Forrest et al. reported that in Afro-Caribbean men, specifically from a Tobagonian population, grip strength increased until 50 years, and decreased thereafter [17]. This trend is further observed across various ethnic groups [18]. McGrath et al., in an analysis of an American population, reported that absolute hand grip strength increased until approximately 25 years of age in men and began to decline at approximately 30 years, while women peaked between 20 to 30 years but maintained strength into mid-life [19]. Mitsionis et al. reported that for every one-year increase in age, grip strength in the dominant hand decreased by 0.61 lbs in men (p=0.015), and by 0.46 lbs in women (p<0.001) in a Greek population [20]. Linear regression confirmed a negative association between age and hand grip strength in the dominant hand for both men and women in our population (p<0.005) (Table 8). Linear regression further confirmed a positive association between height and hand strength in the dominant hand for both men and women in our population. However, while age, weight, height, and BMI were able to predict grip strength in most males (83-85% of the sample), these variables were only able to account for hand strength in an extreme minority of females (<10%) (Table 8). Thus, further research is required to further investigate the variables that predict hand strength in females.

We observed a positive relationship between BMI and hand grip strength in men only, contrasting Alrashdan et al. who reported no correlation in the Saudi Arabian population [10]. Mitsionis et al. observed no association between BMI and hand grip strength in their overall population but reported a moderately negative association among females only [20]. Massy-Westropp et al. reported that higher BMI was weakly related to higher hand grip strength in adults under age 30 and over age 70 in an Australian population but was inversely related to higher hand grip strength between those ages [21].

Our observation that dominant and non-dominant hand grip and key pinch grip in our population were influenced by ethnicity is in keeping with the results reported by McGrath et al. and Ong et al. but contrasts the results from Hossain et al., who found no difference in grip strength among ethnic groups in the Malaysian population [18,19,22]. Previous research has suggested that muscle strength should be interpreted according to ethnicity and that different standards for muscle strength should be applied to multi-ethnic populations [23].

It is logical that an individual’s hobbies and occupation would affect their hand strength. Lim et al., reporting on a Korean population, found that the mean strengths in both hands in participants with more physically demanding occupations were 7.5 kg greater than those of subjects with occupations with low physical demands and 1.9 kg greater than those of subjects with medium physical demands (p=0.001) [11]. We found no significant association between occupational intensity and mean hand strength in our population, contrasting the results by Lim et al. [11]. It is likely that the large number of students, housemakers, self-employed persons, and retired persons in our population influenced this analysis. We recommend that further research account for not only a current occupation but also a person’s hobbies and previous occupation(s).

There are a few limitations to this study. First, we used a convenience sampling method from five towns/cities across Trinidad and Tobago, and therefore subjection selection bias cannot be excluded. However, our relatively large sample size should serve to minimize the effects of any such bias. Furthermore, different instruments were used to assess height, weight, and hand strength at each location, and the possibility of minor instrument errors exists.

This study is relevant to public health experts as hand strength has emerged as an indicator of health-related quality of life and improves cardiovascular disease risk prediction scores [9]. Muscular fitness markers, namely grip strength, have further proven to represent a modifiable risk factor for common mental disorders [24]. This has led to numerous recommendations for the use of grip strength as a screening method in primary and tertiary care [7,9]. This study provides normative/baseline data for the physically healthy adult Trinbagonian population and provides a platform for comparative research and public health strategies.

## Conclusions

This study serves to establish normative hand strength values (i.e., hand grip, palmar grip, pinch grip, and key pinch grip) for the healthy adult Trinbagonian population aged 18-80 years. These values were then compared to international data. We generated fitted linear regression equations to predict dominant hand strength in the Trinbagonian population for all four grips; age, weight, height, and BMI were significant predictors for hand strength in the majority of the male population but were only important predictors for a minority of the female population. Further research to determine better predictive variables specific to the female population is needed. 
